# Person-Centered Integrated Kidney Care Narrative Review: A Model for Reduction of Health Care Carbon Emissions

**DOI:** 10.1177/20543581251391264

**Published:** 2026-02-25

**Authors:** Bhavneet Kahlon, Bhavini Gohel, Sabrina Jassemi, Maoliosa Donald

**Affiliations:** 1 Departments of Medicine and Community Health Sciences, Cumming School of Medicine, University of Calgary, AB, Canada

**Keywords:** chronic kidney disease, carbon emissions, sustainable health care, person-centered care, maladie rénale chronique, émissions de carbone, soins de santé durables, soins axés sur la personne

## Abstract

**Purpose of review:**

Chronic kidney disease (CKD) is a resource-intensive and complex challenge to sustainable health care. The purpose of this narrative review is to describe how person-centered integrated care can support sustainable health care for this population, the strategies currently in place, and the gaps to consider. The findings may support the delivery and improvement of comprehensive support.

**Sources of information:**

Original peer-reviewed articles, website information, and Google Analytics were used to describe the current state of sustainable care strategies for CKD.

**Methods:**

We reviewed the available strategies to support sustainable care through a person-centered approach in Alberta, specifically examining strategies to support care for CKD. These strategies were mapped onto the Centre for Sustainable Healthcare’s four principles of sustainable health care.

**Key findings:**

In Alberta, there are several strategies to support person-centered integrated care. These include resources for health care providers that support prevention and lean service delivery, as well as patient-facing tools to support patient self-care. Person-centered integrated care and sustainability within health care are mutually supportive; however, strategies are often created in silos. There is an opportunity to improve sustainability of kidney care with a comprehensive approach and planning around outcome measures.

**Limitations:**

While these strategies may have significant benefits, there is limited knowledge of direct impacts on outcomes. We used the data that were available and highlighted the need to measure the impact of health care strategies.

## Abrégé

**Motif de la revue:**

L’insuffisance rénale chronique (IRC) est un défi complexe et exigeant en ressources pour la durabilité des systèmes de santé. Cette revue narrative a pour objectifs de décrire comment une prise en charge intégrée et axée sur la personne pourrait contribuer à la durabilité des soins pour cette population, et de présenter les stratégies existantes ainsi que les lacunes à combler. Les conclusions pourraient appuyer la prestation et l’amélioration d’un soutien global.

**Sources:**

Nous avons utilisé des articles scientifiques évalués par les pairs, des renseignements tirés de sites Web et des données de Google Analytics pour décrire l’état actuel des stratégies de soins durables pour l’IRC.

**Méthodologie:**

Nous avons examiné les stratégies mises en place en Alberta pour favoriser une approche de soins durables et axés sur la personne, en examinant plus particulièrement celles qui concernent l’insuffisance rénale chronique. Puis nous les avons alignées sur les quatre principes de durabilité du Centre for Sustainable Healthcare.

**Principales observations:**

En Alberta, plusieurs stratégies appuient une prise en charge intégrée et axée sur la personne. Parmi celles-ci figurent des ressources destinées aux prestataires de soins pour favoriser la prévention et la prestation de services optimisés, ainsi que des outils à l’intention des patients pour les aider à mieux gérer leur santé. En santé, les soins intégrés axés sur la personne et la durabilité se renforcent mutuellement, mais ces stratégies sont souvent élaborées de façon isolée. En soins rénaux, la durabilité pourrait être améliorée grâce à une approche globale et une planification fondée sur les indicateurs de résultats.

**Limites:**

Bien que ces stratégies puissent avoir de grands avantages, les impacts directs sur les résultats demeurent peu documentés. Nous avons utilisé les données disponibles et souligné la nécessité de mesurer l’impact des stratégies de soins.

## Introduction

### Environmental Impact of Health Care

The Canadian health care system is among the world’s most carbon-intensive, responsible for 4.6% of the nation’s total greenhouse gas emissions in 2019.^1^ These emissions, alongside 200 000 tons of other pollutants, contribute to an estimated 23 000 years of life lost annually due to death and disability, exacerbating the very health outcomes the system is designed to protect. Climate-related health impacts increase demand on health care services, further straining a system already under pressure. This is a longstanding issue, as demonstrated in a 2004 study on National Health Service England’s carbon emissions revealed it was responsible for 18 million tons of CO_2_ annually,^2^ accounting for three percent of the United Kingdom’s total emissions. Surprisingly, the largest share did not come from heating and lighting but from clinical care through the procurement of goods and services, which require significant energy for production and delivery.

Canada joined the World Health Organization (WHO) Conference of the Parties 2026 Healthcare Program, committing to net-zero emissions in the health care sector by 2050 and developing climate-resilient, low-carbon systems. The health care sector is responsible for decarbonization to mitigate its environmental impact, safeguard human health, and build resilience to climate change shocks. If health systems are to reach net-zero targets, then new models of care are needed that consider sustainability. The Centre for Sustainable Healthcare in the United Kingdom has laid out four principles of sustainable health care, which may be applied to a new model of care. These include (1) prevention—promoting health and preventing disease, (2) patient self-care—empowering patients to take a larger role in managing their own health, (3) lean service delivery—streamlining to minimize wasteful activities, and (4) low-carbon alternatives—prioritizing treatments and technologies with a lower environmental impact.^3^

There is an opportunity to reduce the carbon footprint of kidney care by addressing the principles of prevention, patient self-care, and lean service delivery during the progressive phases of chronic kidney disease (CKD). Specifically, a comprehensive person-centered integrated care approach can address the complex needs of people living with CKD and improve health care quality by increasing access to preventive services, reducing unnecessary service utilization such as hospitalizations, and decreasing care fragmentation. The purpose of this narrative review is to describe how person-centered integrated care can support sustainable health care for this population, the strategies currently in place, and the gaps to consider.

### Person-Centered Integrated Care and CKD

Person-centered integrated care is described by the WHO as health services that are managed and delivered such that patients receive coordinated care across different levels of the health system that address the continuum of preventive and curative services according to patients’ needs throughout the life course. The intended purpose of this approach is to optimize equity in access, quality, responsiveness and participation, efficiency, and resilience.^4-6^

The global burden of kidney disease is overwhelming, with an estimated 850 million people worldwide currently affected.^7^ This number is expected to increase and it is expected that by 2030, 5.4 million people worldwide will be receiving kidney replacement therapies (KRTs).^7^ Diagnosing and treating CKD early not only slows disease progression, but can also address cardiac complications and avoid hospitalizations. In Canada in 2023, almost 30 000 people were on dialysis.^8^ This concern is further compounded by the resource intensity required to provide care for people with kidney diseases. For example, patients with chronic CKD in the United Kingdom have hospital stays that are on average 35% longer than patients who do not have CKD.^7^ In Canada, patients with CKD have similar negative outcomes to patients with cancer for mortality, hospitalization, and likelihood of placement into long-term care.^9^ It has also been shown that, compared with patients without kidney disease, patients in Canada on dialysis tend to undergo more surgeries, have longer post-operative hospital stays, and are much more likely to be discharged to a facility requiring 24-hour care.^10^ In addition, hemodialysis is known to be a water and energy intensive therapy, and it has been estimated that patients on hemodialysis three times per week have a carbon footprint that is seven times higher than the average patient in UK health care.^11^

**Figure 1.**
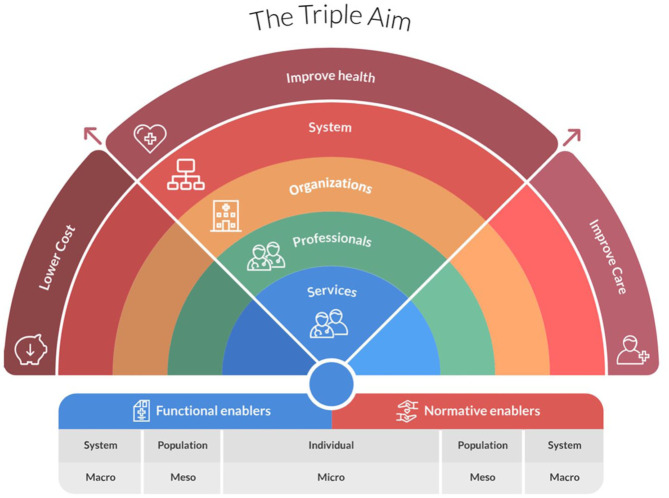
Rainbow model of integrated care.^14^

Delivering person-centered integrated care for patients with CKD can be challenging as it can be incompatible with how health care is delivered at the macro (systems), meso (organizational), and micro (clinical) levels (Figure 1).^12^ These domains, based on the rainbow model of integrated care (RMIC) framework, considers the domains of the Triple Aim in its development.^13^ However, there are potential strategies that can support integrated care. Such strategies can improve patient care directly (eg, self-management support), change health care provider practices (eg, clinical guidelines), and transform organizations (eg, care pathways and clinical information systems).

Assessing the outcomes of a complicated, multi-faceted intervention such as person-centered care is challenging. A meta-analysis of randomized controlled trials of person-centered integrated care for CKD found that person-centered integrated care may lead to fewer hospitalizations and improved blood pressure control.^5^ Although the impact on kidney function and KRT was uncertain, with little effect on mortality or quality of life, fewer hospitalizations would translate to lower resource utilization and thus one could infer, lower-carbon footprint. A more recent study from Thailand found that an integrated care model was effective in delaying CKD progression,^15^ which would translate to fewer patients requiring KRT or delayed KRT starts, meaning lower resource utilization.

## Methods

Although optimizing the environmental sustainability of health care is not an explicitly stated goal of person-centered integrated care, many of the features of this approach fit into the framework laid out by the Centre for Sustainable Healthcare and thus implementing integrated care strategies could potentially impact the carbon footprint of health care. We reviewed the available strategies to support sustainable care through a person-centered approach in Alberta, specifically examining strategies to support care for CKD. We would like to highlight four strategies available to health care providers, patients, and caregivers in Alberta, Canada that address person-centered integrated care and the principles of sustainable health care. These include the CKD pathway, My Kidneys My Health, the conservative kidney management (CKM) pathway, and a provincial electronic medical record (EMR). To describe the development, uptake, and potential influence of these strategies on sustainable outcomes, our sources of information included original peer-reviewed articles, website information, and Google Analytics. While there are studies that reference the impact of certain strategies (eg, CKD pathway^16^ and My Kidneys My Health^17^), there is a gap in literature that examines the impact of using these strategies together.

## Review

**Figure 2.**
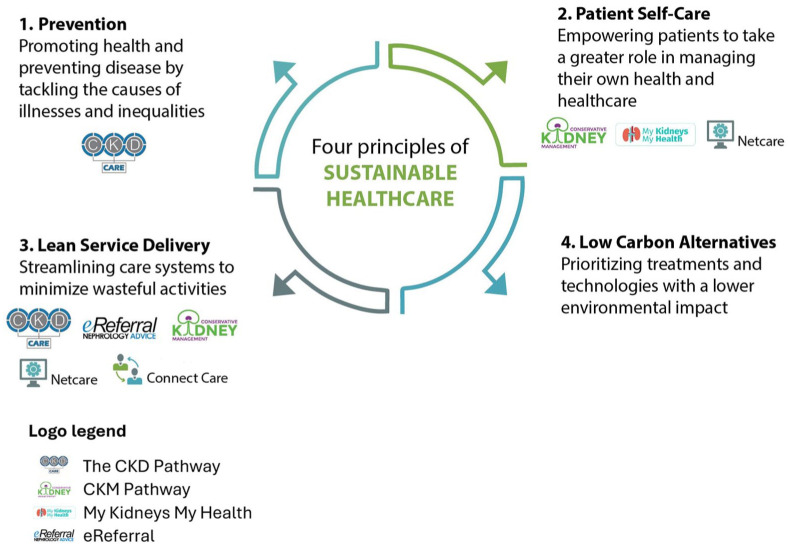
Principles of sustainable health care, adapted to include logos of strategies available in Alberta.

Each strategy (Table 1) addresses one or more of the principles of sustainable health care, as shown in Figure 2.

**Table 1.** Sustainable Health Care Strategies in Alberta.

Strategy	Program description	Sustainable outcomes
CKD pathway (*prevention and lean pathways*) https://ckdpathway.ca/	An interactive tool that provides guidance and supporting information to primary care providers to aid in the diagnosis, management, and referral of adults with CKD. Based on current evidence-based clinical practice guidelines	Enhanced access to suitable services and prompt care Increased health care provider competencies for CKD diagnosis and care Identification, treatment and management of CKD and relevant comorbidities
My Kidneys My Health (*patient self-care*) https://mykidneysmyhealth.com/	An interactive tool co-developed with patients to assist patients and caregivers in learning about CKD and actively managing their CKD. Based on current evidence-based clinical practice guidelines and expert advice	Support of patient empowerment, through educating patients on CKD and its management Addressing social determinants of health—finances, work, and education
Conservative kidney management pathway (*patient self-care and lean pathways*) https://www.ckmcare.com/	A pathway for patients and health care professionals regarding quality of life, symptom management, and living well without dialysis. Guidelines co-developed with patients and health care professionals based on comprehensive literature searches and extensive stakeholder engagement	Reduction in unnecessary hospitalizations and emergency visits Avoiding inappropriate life-sustaining care that does not align with patients’ values Evidence-informed decision-making tools
Provincial EMR (*lean pathway and patient self-care*)	Electronic medical and health records are used as a secure method of storing and sharing information. For example, Alberta Netcare and Connect Care support the flow of information between health care providers and patients and their health care providers. eReferral is an online referral management system	Coordinated care across the continuum Reduction in the duplication of care and investigation Data sharing between patient and caregivers Collaboration across specialities

### Description of Sustainable Health Care Strategies

The first sustainable health care strategy is called the “CKD Pathway,”^18^ developed to translate the Kidney Disease: Improving Global Outcomes (KDIGO) Clinical Practice Guidelines for the Evaluation and Management of CKD into clinical practice at the point of care. The pathway promotes early diagnosis, appropriate pharmacotherapy and lifestyle recommendations, and timely nephrology referrals.^19^ These values are incorporated into the CKD pathway. Historically, limited pharmacotherapy options were available for slowing the progression of CKD; however, in recent years, many new agents are available to slow down the progression of CKD and early recognition can lead to improved patient outcomes.^7^ For example, there is good evidence that medications, such as renin-angiotensin-aldosterone system inhibitors, sodium-glucose cotransporter 2 (SGLT2) inhibitors and glucagon-like peptide-1 (GLP-1) receptor agonists, such as Ozempic, slow down the progression of CKD.^20-29^ The CKD pathway provides health care providers, particularly primary care providers, with prescribing practices on these agents with the goal to slow down the progression of CKD to end-stage kidney disease, thereby reducing the need for much more resource-intensive therapies such as hemodialysis. It also addresses the concept of lean service delivery by providing health care providers with point of care diagnosis and referral criteria to nephrologists, which avoids unnecessary health care and resource utilization. The pathway additionally provides guidance on the frequency of testing and which tests to order for patients, which can reduce the waste associated with unnecessary testing. Since the launch of the CKD pathway, the website has been accessed by more than 150 000 users globally (2016-2024). Following the implementation of the CKD pathway in Alberta, an increase in albumin-to-creatinine ratio (ACR) testing (guideline concordant care) for relevant patients was observed in a retrospective cohort study.^16^

The second strategy is a comprehensive and tailored website, “My Kidneys My Health” which was co-created with patients to support self-management in early-stage CKD.^30,31^ This self-care tool provides guidance and information to patients and their caregivers, which can potentially lead to multiple environmental sustainable outcomes. For example, accessing credible information to manage their condition at home may reduce health care visits and thus reduce the associated carbon footprint and resource utilization required for tests, transportation, and facilities. The website can promote preventative self-care behaviours through personalized patient education and recommendations related to medication, lifestyle and diet. Adherence to these recommendations can lead to better disease control and hopefully slow down the progression to more severe disease. My Kidneys My Health was tested with patients and found to have high acceptance, supported confidence, and influenced changes in behavior related to diet, exercise, and medications.^32^ My Kidneys My Health has seen an increase in users each year, with more than 17 000 users since its launch (2021). Health care providers can hold an influential role in the implementation and dissemination of tools such as My Kidneys My Health in clinical practice.

The third strategy is the “Conservative Kidney Management”^33^ (CKM) pathway, a tool for providers and patients that focuses on CKM as a treatment option for kidney failure to improve quality of life, symptom control, and living well without dialysis in those who are unlikely to benefit from dialysis. With its basis in the principles of person-centered care, the CKM pathway presents an opportunity to support both patients and health care providers in shared decision-making. The CKM pathway provides a sustainable strategy to reduce inappropriate use of resource-intensive modalities through educational and decision supports. As with My Kidneys My Health, the implementation of the CKM pathway is strengthened by the adoption of various types of health care providers.^34^ This comprehensive strategy has also supported coordinated and accessible self-management tools for more than 25 000 website users since its launch (2016).

Finally, provincial EMRs can be used to improve sustainability in many ways. In Alberta, several EMR systems are used in the inpatient and outpatient settings (e.g., Alberta Netcare, and Connect Care). All acute care sites in Alberta have adopted the use of one EMR. This electronic information system has the potential to reduce redundant testing and referrals, improve communication between care providers and patients, as well as reduce unnecessary paper wastage. For instance, if a health care provider orders a test or investigation that has been already ordered by another care provider, the system will alert the user which can reduce unnecessary duplications. Referrals made to specialists are visible to all care providers, again reducing duplication. Patients can also access their own clinical information such as test results, appointment notifications and reminders, and medication list. This can enhance patient self-care and lead to less missed appointments. Another area is the provincial electronic referral (eReferral) system, which allows health care providers to obtain specialist advice without the need for a formal consult in all cases. In a study examining implementation of e-consult services in Canada, Alberta’s eReferral service was the largest with 1446 participating primary care providers and a low implementation burden by integrating the function into an existing and familiar platform.^35^ This online consultation service can reduce the carbon footprint associated with a specialist visit, including the additional testing often required and potential transport to see a specialist when the patient could be appropriately managed by the referring physician. In general, the use of EMRs is likely positive; however, unknowns regarding the net impact of energy intensive features, such as embedded artificial energy, remain.

These examples support how person-centered integrated strategies can be implemented within the framework of sustainable health care to reduce the carbon footprint of kidney care globally.

## Discussion

Person-centered integrated care, when implemented effectively, offers a model that aligns well with the principles of sustainable health care, ensuring both equity and access to necessary services. This approach to health care system integration ties into sustainability, offering a pathway toward improving the system’s long-term viability. For example, in kidney care, integration plays a critical role in enhancing sustainability by ensuring services are interconnected and aligned with broader health system goals. However, many health care programs have traditionally been developed in silos, creating opportunities to modify and better align these initiatives, making sure they contribute to both system integration and carbon mitigation. As such, this narrative review identifies the gaps that exist in sustainable health care for this population.

Currently, health care programs tend to operate in isolation, with minimal integration into existing services, in addition to lack of coordination of strategies from micro to macro-levels. There is a need for a more comprehensive approach to assess the health care landscape, identify gaps, and ensure that new programs are introduced in a way that adds value. Without this thoughtful planning, there is a risk of duplicating efforts, failing to deliver value-based care, or exacerbating the health care system’s already significant carbon footprint, all while trying to meet the needs of patients. Sustainability and scale-up of interventions must also be considered. For example, despite the initial uptake of eReferral in Alberta, trends show irregular usage of the service.^35^ While EMRs enhance integrated care through supporting coordination of services and communication between health care providers and patients, in Alberta Connect Care is currently available within Alberta Health Services clinics (mainly specialty and acute care) and is not the main EMR for providers within the community (i.e., primary health care physicians, and pharmacists). For patients with CKD and multimorbidity, most of their day-to-day care is managed within the community setting.

## Limitations

Funding and policy support is essential in supporting research to identify the evidence to support person-centered integrated care and sustainability. Currently, there is limited knowledge regarding the direct outcomes of some initiatives, including patient empowerment, coordinated care, and evaluating emissions from integrated care. The described strategies may have significant intangible benefits but tracking their concrete impacts can be challenging. We incorporated all available data to describe the strategies and potential benefits. This challenge highlights a need to measure the impact of health care strategies. Given the carbon intensity of today’s health care systems there is a pressing need, more than ever, for integration and lean pathways that reduce unnecessary resource use while delivering high-quality care.

Person-centered integrated care is not only a key concept but also a driver of change toward leaner, lower-carbon health care systems. This model places a strong emphasis on patient empowerment and prevention, focusing on the entirety of a patient’s health journey from prevention and disease detection to treatment. The potential for integrating care in this way is vast, but each program must be carefully evaluated to ensure it meets patient outcomes while delivering positive social and environmental impacts. Crucially, it must do so without imposing unnecessary financial burdens on the health care system.

## Conclusion

Person-centered integrated care and sustainability within health care are mutually supportive. With the growing global demand for kidney care, leveraging this approach to improve the sustainability of kidney care is an opportunity that warrants further exploration. To maximize its potential, it is crucial to focus on providing comprehensive care and identifying the right outcome measures to ensure that the desired results are effectively achieved.
